# Vertigo as a Predominant Manifestation of Neurosarcoidosis

**DOI:** 10.1155/2015/397046

**Published:** 2015-04-02

**Authors:** Tasnim F. Imran, Sobia Nizami, Igor Eyzner, Neena Mirani, Tanzib Hossain, Robert Fede, Eugenio Capitle

**Affiliations:** ^1^Department of Medicine, New Jersey Medical School Rutgers, The State University of New Jersey, 185 South Orange Avenue, Newark, NJ 07103, USA; ^2^Department of Pathology and Laboratory Medicine, New Jersey Medical School Rutgers, The State University of New Jersey, 185 South Orange Avenue, Newark, NJ 07103, USA; ^3^Division of Allergy, Immunology, and Rheumatology, New Jersey Medical School Rutgers, The State University of New Jersey, 185 South Orange Avenue, Newark, NJ 07103, USA

## Abstract

Sarcoidosis is a granulomatous disease of unknown etiology that affects multiple organ systems. Neurological manifestations of sarcoidosis are less common and can include cranial neuropathies and intracranial lesions. We report the case of a 21-year-old man who presented with vertigo and uveitis. Extensive workup including brain imaging revealed enhancing focal lesions. A lacrimal gland biopsy confirmed the diagnosis of sarcoidosis. The patient was initially treated with prednisone, which did not adequately control his symptoms, and then was switched to methotrexate with moderate symptomatic improvement. Our patient had an atypical presentation with vertigo as the predominant manifestation of sarcoidosis. Patients with neurosarcoidosis typically present with systemic involvement of sarcoidosis followed by neurologic involvement. Vertigo is rarely reported as an initial manifestation. This case highlights the importance of consideration of neurosarcoidosis as an entity even in patients that may not have a typical presentation or systemic involvement of disease.

## 1. Introduction

Sarcoidosis is a granulomatous disease of unknown etiology that can involve several organ systems. Neurological symptoms are not common and rarely occur initially without systemic involvement [1, 2]. We describe the case of a 21-year-old man presented with vertigo as the initial manifestation of sarcoidosis.

## 2. Case Presentation

We report the case of a 21-year-old African American man with no significant past medical history who presented with dizziness and right eye pain for two weeks. He reported swelling, erythema, blurred vision, tearing, and light sensitivity. He also developed fatigue, dizziness, light headedness, and vertigo. The vertigo had developed gradually over two months. He described it as a spinning sensation when sitting still, which was exacerbated by movement. He denied tinnitus, hearing loss, fever, headache, nausea, vomiting, neck pain, episodes of loss of consciousness, focal weakness, sensory loss, or seizures.

On physical examination, he was afebrile and normotensive, with pulse rate 74 beats/minute and BMI 21.7 kg/m^2^. The right eye had conjunctival injection and periorbital edema. Visual acuity was 20/30 in both eyes. Pupillary reflexes and extraocular movements were intact. Systemic examinations for cardiovascular, respiratory, musculoskeletal, and gastrointestinal examinations were within normal limits. No hepatomegaly was noted. Ear, nose, and throat exam was normal, with no hearing loss, parotid enlargement, or sinonasal lesions and no lymphadenopathy. Neurologic examination revealed a swaying gait, and the patient was unable to perform heel-to-toe and tandem walk. Romberg's test was negative. There was no dysmetria and no ataxia on finger-to-nose and heel-to-shin testing.

A complete blood count, chemistry panel, and urinalysis were within normal limits. Erythrocyte sedimentation rate was 15 mm/hour, CRP 1 mg/L. On further testing, the patient had a nonreactive rapid plasma reagent, Quantiferon gold test, HIV rapid screen, hepatitis panel, Monospot test and Lyme western blot, HLA-B27, anti-cardiolipin antibody, antinuclear antibodies, double-strand DNA, Smith, RNP antibodies, and angiotensin converting enzyme (ACE). Lumbar puncture revealed no blood, 6 WBCs with a lymphocytic predominance, ACE level of <3 units, and protein 35 mg/dL.

**Figure 1:**
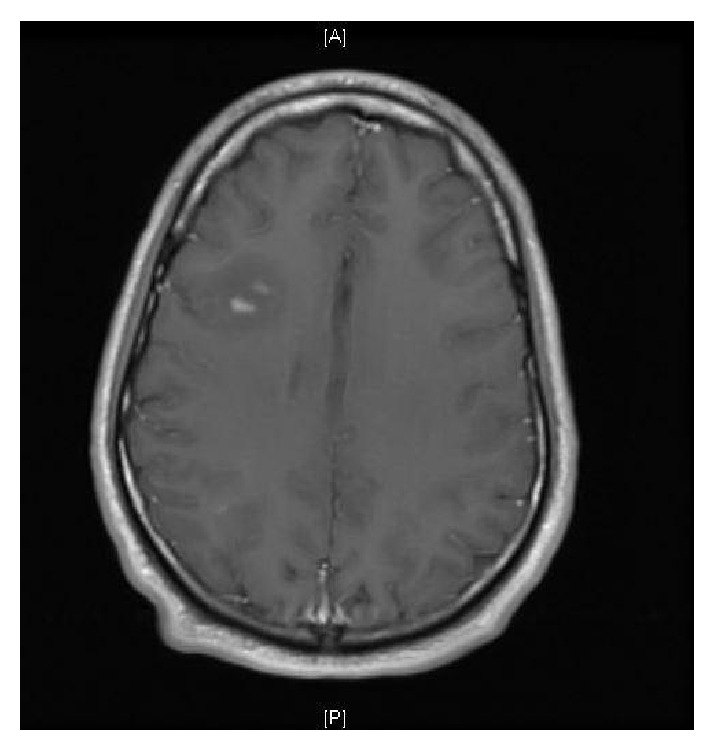
A T1-weighted postgadolinium magnetic resonance image demonstrating two nodular foci of enhancement with surrounding vasogenic edema.

**Figure 2:**
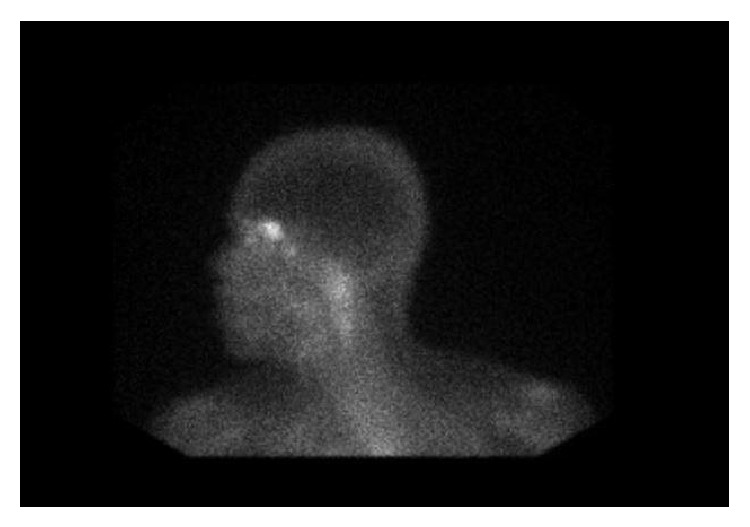
This image from the gallium scan demonstrates symmetric increased uptake in the lacrimal glands and the parotid gland. Increased gallium uptake is suggestive of an inflammatory process such as sarcoidosis.

**Figure 3:**
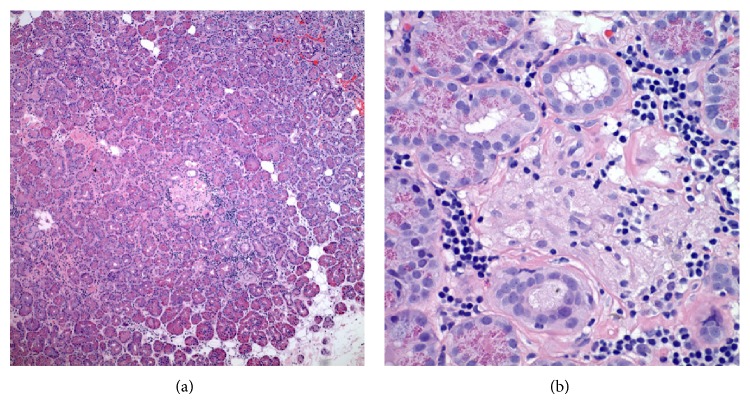
(a) Hematoxylin and eosin stain at 100x magnification, showing a lacrimal gland section with small nonnecrotizing granulomas. (b) Hematoxylin and eosin stain at 400x magnification, showing a small nonnecrotizing granuloma with epithelioid cells and lymphocytes.

The chest radiograph was normal, with no mediastinal lymphadenopathy. 12-lead electrocardiogram demonstrated normal sinus rhythm. Due to patient's persistent vertigo, a computed tomography (CT) scan of the head was performed, which showed a focal low density in the right frontal lobe suggestive of vasogenic edema. Follow-up magnetic resonance imaging (MRI) scan of the brain confirmed this finding and showed additional foci of enhancement in the frontal and parietal subcortical white matter (Figure 1). A gallium scan demonstrated symmetric moderately increased uptake in the lacrimal glands bilaterally and moderately increased uptake in the parotid glands (Figure 2). Given suspicion for sarcoidosis, biopsy of the right lacrimal gland was performed. Small, nonnecrotizing epithelioid granulomas were visualized on biopsy (Figures 3(a) and 3(b)). The specimen was negative for acid-fast bacilli and fungi.

Based on imaging and biopsy findings, the patient was diagnosed with neurosarcoidosis and uveitis. Therapy with prednisone was initiated. Despite treatment, his vertigo persisted, and he developed a recurrence of anterior uveitis four months later. Methotrexate was added and prednisone was discontinued. Repeat brain imaging demonstrated resolution of the lesions in the frontal cortex four months after treatment with methotrexate. Ocular disease also resolved, with partial improvement of vertigo symptoms. Methotrexate 15 mg weekly was continued with regular follow-up.

## 3. Discussion

Sarcoidosis is a granulomatous disease that affects multiple organ systems. In the United States, African Americans have a 10 times higher incidence as compared to Caucasians. The prevalence ranges from 10 to 40 per 100,000, with a mortality of 1 to 5% [1, 3].

Environmental and genetic factors have been implicated in the pathogenesis of sarcoidosis. It may result from exposure of a genetically susceptible host to specific environmental factors that stimulate a Th-1 immune response [2]. Environmental factors include infections such as* Mycobacterium tuberculosis* and* P. acnes* as well as other exposures such as pesticides, silica, or metal dusts [2]. The Th-1 response produces cytokines including interferon-gamma, interleukins 2, 6, 12, and 16 [4]. The cytokines recruit additional phagocytes and T cells, leading to the aggregation of phagocytes into epithelioid cells and giant cells. The hallmark of sarcoidosis is the noncaseating granuloma, which consists of activated macrophages and CD4-helper T lymphocytes [2]. Granulomas are the source of ACE, which is found to be elevated in some cases [4].

Neurologic complications occur in only 5% of all patients with sarcoidosis [2]. Early involvement of the nervous system is rare but was the case of our patient [5]. Clinical manifestations of neurosarcoidosis fall into several syndromes. Most commonly, patients present with cranial mononeuropathies, with facial nerve palsy being the most prevalent [1]. Otolaryngologic manifestations are identified in 10% of patients [6]. Ear involvement and vertigo are also rarely encountered. They are seen in the setting of vestibulocochlear nerve involvement; therefore, patients present with sensorineural hearing loss in association with vertigo and gait ataxia [7].

Vertigo is a symptom of dysfunction of the vestibular system. The initial evaluation of vertigo includes an ear examination and bedside hearing tests, positional maneuvers such as the Dix-Hallpike maneuver, and audiometry and caloric testing. Positive findings on these tests indicate a peripheral vestibular lesion, such as benign positional vertigo [8]. Stroke syndromes are the main etiology of central vertigo. Intracranial mass lesions are not associated with isolated vertigo in the absence of focal neurological findings, which makes our patient's presentation unique. Imaging is indicated for patients suspected to have a central lesion, stroke, and a prolonged history of vertigo with significant functional impairment [7]. In our patient, there was no hearing loss or other findings to suggest peripheral cranial nerve eighth involvement; however, he continued to have persistent symptoms; therefore, imaging was pursued.

Lumbar puncture in sarcoidosis may demonstrate an elevated CSF protein and a mild-to-moderate pleocytosis with lymphocytic predominance or may be normal in a third of patients [9, 10]. Decreased glucose levels may occur in one-fifth of patients [10]. Our patient's CSF showed lymphocytic predominance with normal protein.

There are several imaging modalities for diagnosis, staging, and management of patients with sarcoidosis. MRI with contrast is the imaging modality of choice for neurosarcoidosis. Whole body gallium scan or fluorodeoxyglucose PET scan may aid in finding sites for biopsy to confirm diagnosis [11]. Neurosarcoidosis remains a diagnostic challenge because these imaging modalities are sensitive but not specific. Additionally, neurosarcoidosis can vary greatly in appearance on imaging [12]. The most common finding is the involvement of the leptomeninges seen as nodular or linear enhancement along the brain's contours extending into the cortical sulci [9, 12]. However, this can also be seen with meningeal carcinomatosis, lymphoma, leukemia, tuberculosis, and fungal meningitis [12]. Enhancement of the cranial nerves or the hypothalamic-pituitary axis may also be noted, although involvement of cranial nerves on imaging may correlate poorly with the clinical neurologic deficit [9, 12]. For example, although cranial nerve II (optic nerve) most often shows enhancement on imaging, it is cranial nerve VII (facial nerve) that tends to show the clinical neurologic deficit [12]. Brain MRI with gadolinium enhancement usually reveals enhancement of the eighth cranial nerve [13]. On biopsy, noncaseating granulomas are the classic finding in sarcoidosis but are only diagnostic in the presence of clinical features and after exclusion of tuberculosis, fungus, malignancy, or other causes of granulomas [2].

There are no randomized controlled trials in the treatment of neurosarcoidosis, but general consensus dictates that patients with active disease should be treated with corticosteroids. Corticosteroid dose and duration of therapy are guided by disease severity and response to therapy [2, 12]. Most patients are prescribed prednisone; those with severe or incapacitating disease may respond to intravenous methylprednisolone for a few days, followed by oral prednisone. Doses are higher than those advised for patients with localized sarcoidosis. Prednisone 1 mg/kg/day is usually recommended [2]. A response to therapy is expected within six to eight weeks [4].

Alternative therapies are considered for patients with refractory disease. A specific therapy is often chosen based on cost, availability, and side effect profile. Mycophenolate mofetil, azathioprine, methotrexate, cyclophosphamide, cyclosporine, and cladribine have been used. Methotrexate and azathioprine may be preferred due to safety and efficacy considerations. Methotrexate is easily tolerated with minimal side effects; however, liver function should be monitored. The response rate is about 60%, similar to other medications [2]. Our patient was initially started on prednisone but due to a poor response was switched to methotrexate.

Infliximab has been shown to be effective in a few cases. Radiation therapy may be considered in patients with CNS disease that is refractory to medical treatment [2]. Although methotrexate and mycophenolate mofetil are often selected after corticosteroid failure, other options include anti-TNF alpha agents and intravenous cyclophosphamide [14–16]. Anti-TNF*α* agents have shown encouraging results in the treatment of ocular sarcoidosis but further controlled studies are needed to elucidate their role [17].

In conclusion, our patient had a rare presentation of vertigo as the predominant manifestation of sarcoidosis along with parenchymal brain lesions, without any other focal neurologic findings, evidence of eighth cranial nerve involvement, or systemic features. Most patients with neurosarcoidosis tend to have systemic involvement prior to neurological symptoms. This poses a diagnostic dilemma for the clinician. This case highlights the importance of recognition of this entity and consideration of neurological involvement of sarcoidosis even in patients who may not have typical manifestations or systemic involvement.
